# Morphological and Ultrastructural Characterization of Antennal Sensilla and the Detection of Floral Scent Volatiles in *Eupeodes corollae* (Diptera: Syrphidae)

**DOI:** 10.3389/fnana.2021.791900

**Published:** 2021-12-16

**Authors:** Wan-Ying Dong, Bing Wang, Gui-Rong Wang

**Affiliations:** ^1^State Key Laboratory for Biology of Plant Diseases and Insect Pests, Institute of Plant Protection, Chinese Academy of Agricultural Sciences, Beijing, China; ^2^Shenzhen Branch, Guangdong Laboratory for Lingnan Modern Agriculture, Genome Analysis Laboratory of the Ministry of Agriculture, Agricultural Genomics Institute at Shenzhen, Chinese Academy of Agricultural Sciences, Shenzhen, China

**Keywords:** antenna, sensilla, odorant receptor neuron, scanning electron microscopy, transmission electron microscopy, single sensillum recording, methyl eugenol

## Abstract

The olfactory sensing system of the syrphid fly *Eupeodes corollae* is essential in pollination and prey localization, but little is known about the ultrastructural organization of their olfactory organs. In this study, the morphology, distribution, and ultrastructural organization of antennal sensilla of *E. corollae* in both sexes were observed by scanning electron microscopy (SEM) and transmission electron microscopy (TEM). Neuronal responses of a subtype of sensilla basiconica to floral scent compounds were recorded by single sensillum recording (SSR). Ten morphological types, including Böhm bristles, sensilla chaetica, microtrichiae, sensilla trichodea, sensilla basiconica, sensilla clavate, sensilla coeloconica, sensilla styloconica, sensilla placodea, and sensory pit, were identified. Except for Böhm bristles and sensilla chaetica, which were distributed on the scape and pedicel of *E. corollae* antennae, innervated sensilla were densely distributed on the flagellum, a vital sensory organ. Further, observing ultrastructural organization showed that the sensilla trichodea, basiconica, and clavate are single-walled with multiple nanoscale pores perforating the cuticle. Sensilla coeloconica are double-walled and have no wall pores, but instead, have longitudinal grooves along with the pegs. Sensilla chaetica, Böhm bristles, and microtrichiae did not have wall pores on the cuticle or sensory cells at the base. The SSR results indicated that neuron B housed in the subtype of sensilla basiconica I (SBI) mainly responded to methyl eugenol and other aromatic compounds. Overall, our results provide valuable information to understand the morphology and ultrastructure of antennal sensilla from *E. corollae*. These findings are beneficial for the studies of the neuronal function map of olfactory sensilla and for determining evolutionary relationships in Diptera.

## Introduction

The insect olfactory system allows sensitive detection and precise discrimination of relevant odor cues from natural surroundings (Bruce et al., 2005; Renou and Anton, 2020). These cues include semiochemicals released by food sources, oviposition sites, predators, or competitors and also pheromones emitted from conspecifics (Fleischer et al., 2018; Renou and Anton, 2020). Various volatile organic compounds are detected by odorant receptor neurons (ORNs) housed in the sensilla, hair-like structures that extend from the insect cuticle with multiple cuticular pores on the antennae and maxillary palps (Steinbrecht, 1997; Keil, 1999). Odorants are thought to penetrate through the pores of the sensilla walls into the sensillum lymph and are transferred by odorant-binding proteins (OBPs) toward the dendrites of ORNs and then activate the odorant receptors (ORs) to generate action potentials (Leal, 2013). Subsequently, electrical signals are transmitted through the axons of ORNs and converge to the central nervous system (Wilson, 2013). The higher olfactory center reintegrates and processes this information and finally causes the insect to produce corresponding behavioral responses (Bates et al., 2020).

Odorants are initially discriminated by dedicated olfactory sensilla located on insect antennae (Ghaninia et al., 2014; Keesey et al., 2015). Generally, sensilla involved in olfaction occur in three major morphological types, trichoid, basiconic, and coeloconic (Venkatesh and Singh, 1984). The sensilla types, however, differ not only in micromorphological and anatomical structure but also in their functional responses to the activation of receptors and neurons. Sensilla trichodea in many insects, such as flies and moths, are mainly tuned to detect pheromone components (Wang et al., 2016; Chang et al., 2017; Liu S. et al., 2020; Khallaf et al., 2021). However, some sensilla trichodea detect other chemicals (Soni et al., 2019). For example, in the tsetse fly *Glossina morsitans* (Diptera: Glossinidae), ORNs housed in trichoid sensilla respond to a wide diversity of chemicals, such as 1-octen-3-ol, 2-heptanone, isoamyl acetate, and methyl laurate. Sensilla basiconica usually responds to plant volatiles, including many alcohols, aldehydes, esters, ketones, and carbon dioxide (de Bruyne et al., 2001; Keesey et al., 2015; Cui et al., 2018; Mansourian et al., 2018). ORNs in coeloconic sensilla are tuned to specific chemosensory stimuli, including acids, aldehydes, ammonia, putrescine, and water vapor (Yao, 2005; Prieto-Godino et al., 2016, 2017). Some sensilla coeloconica also respond to a range of temperatures and humidity (Ruchty et al., 2010; Schneider et al., 2018).

In addition to these common sensilla, other types of sensilla are found on Dipteran insect antennae. Sensilla styloconica are distributed on the antennal pedicel of Anthomyiidae (Ross, 1992) and Calliphoridae (Sukontason et al., 2004; Hassan et al., 2013) and the antennal flagellum of Tephritidae (Arzuffi et al., 2008; Bisotto-De-Oliveira et al., 2011), the function of which is supposed to hygro- and thermoreception. Sensilla auricillica was observed on the antennal flagellum of four species of Oestridae (Zhang et al., 2016). Moreover, Shanbhag et al. (1995) found sensory sacculus on the antennal flagellum of *Drosophila melanogaster*. The same structures were also found on the antennal flagellum of *Triceratopyga calliphoroides* (Zhang et al., 2014), *Fannia canicularis*, and *F. scalaris* and were defined as a multichambered invagination stretching into the cavity of the antennal funiculus containing different types of sensilla (Zhang et al., 2013a).

Similarly, sensory pits, single-chambered invaginations containing a cluster of sensilla with a fringe of microtrichiae around the edge, have been found on the antennae of flies from Fanniidae (Wang et al., 2012; Zhang et al., 2013a), Anthomyiidae (Honda et al., 1983; Ross, 1992), Muscidae (Sukontason et al., 2004; Smallegange et al., 2008; Tangtrakulwanich et al., 2011), Sarcophagidae (Liu et al., 2016; Pezzi et al., 2016), Oestridae (Zhang et al., 2012a,^2016^; Liu et al., 2015), Calliphoridae (Setzu et al., 2011; Zhang et al., 2013b,^2014^), Syrphidae (Henderson and Wellington, 1982; Jia et al., 2019), and Glossinidae (Isaac et al., 2015). Numerous sensilla gathered in the sensory sacculus and sensory pits can increase the contact area between the sensilla and the odor, improve the efficiency of the sensilla to capture the odor, and enhance the olfactory sensitivity of the antennae (Hunter and Adserballe, 1996; de Bruyne et al., 2001; Zhang et al., 2013b). The morphology and function of these different types of sensilla may be the result of the long adaptation of insects to the surrounding environment. Therefore, determining external morphology and fine structure of the sensilla will help in studies of comparative morphology and reveal mechanisms of olfactory recognition and evolution and adaptation in insects.

Larval hoverflies are a natural enemy of aphids that can significantly suppress aphid populations (Wotton et al., 2019). As adults, hoverflies are important pollinators (Baldock et al., 2019; Rader et al., 2020). In flowering plants, the aromatic compounds aldehydes, alcohols, ethers, and esters alone or in combination with some monoterpenes alcohols are often identified as floral volatiles perceived by and attractive to flower visitors or pollinators, such as bees, syrphid flies, butterflies, and moths (Dobson, 2006; Dötterl and Vereecken, 2010; Primante and Dötterl, 2010; Zito et al., 2019). Representative floral volatiles includes 2-phenylethanol, methyl salicylate, limonene, eugenol, and methyl eugenol, all reported to be detected by and an attractant for syrphid flies (Zhu and Park, 2005; Benelli et al., 2017; Li et al., 2020). In addition, inflorescence scents involving phenylacetaldehyde, pyranoid linalool oxide, methyl salicylate, dimethyl salicylate, linalool, and octyl acetate were recently reported as electrophysiologically active compounds for Syrphidae (Primante and Dötterl, 2010; Braunschmid et al., 2017). These findings emphasize that floral volatiles, especially aromatic compounds, are essential for attracting pollinating hoverflies. But the olfactory tools used by hoverflies to detect complicated floral cues have not been investigated (Braunschmid et al., 2017).

The hoverflies, *E. corollae* (Diptera, Syrphidae), are a dual service provider and a widely distributed species in the agricultural ecosystem of north-eastern China (Pekas et al., 2020). In this study, we describe the antennal sensilla morphology and distribution in *E. corollae* using SEM. The antennal sensilla in male and female *E. corollae* were classified into ten types according to their shape and morphological features. The ultrastructural organization and the neuronal numbers of seven types of sensilla were further investigated by TEM. Then, we recorded responses of a single neuron in the subtype of sensilla basiconica I (SBI) to ten floral compounds on the antennae of male and female *E. corollae* using single sensillum recording (SSR) technology. Our study provides useful information in the aspect of the morphological types and ultrastructure of the antennal sensilla and helps to understand the molecular mechanisms of olfactory perception at the peripheral nervous system in *E. corollae*.

## Materials and Methods

### Insect Rearing

Adults *E. corollae* were collected from the Langfang Experiment Station of the Institute of Plant Protection, Chinese Academy of Agricultural Sciences (CAAS), Langfang, Hebei province, China (116.60°E′, 39.50°N′). *E. corollae* was reared in our laboratory with *Acyrthosiphon pisum* (Hemiptera: Aphididae) at the larval stage and pollen and 10% honey solution at the adult stage at 25 ± 1°C, 60 ± 5% relative humidity and under a photoperiod of 14 h light: 10 h dark. After eclosion, females and males were processed for observation with SEM and TEM. Three- to four-day-old virgin females and males were used for electrophysiological recordings.

### Scanning Electron Microscopy

Antennae of male and female adult hoverflies were excised from the base under a stereomicroscope and sonicated in 70% ethanol in 1.5 ml centrifuge tubes for 10 s in an ultrasonic bath (250W) to wash away impurities on the surface of the antennae. Subsequently, the specimens were dehydrated in ethanol solutions (80, 90, and 100%) for 5 min each. After drying in a carbon dioxide critical point drier (LEICA EM CPD030), the specimens were mounted on aluminum stubs with double-sided conductive adhesive, coated with gold in an ion sputtering device (HITACHI MC 1000), and stored in a desiccator until use. The preparations were examined with a HITACHI SU8010 scanning electron microscope (Hitachi, Tokyo, Japan) at the Electronic Microscopy Centre of the Institute of Food Science and Technology, CAAS (Beijing, China). Sensilla types in this study were classified according to the previous references (Bisotto-De-Oliveira et al., 2011; Pezzi et al., 2016; Hore et al., 2017).

### Transmission Electron Microscopy

Antennae were dissected from newly emerged male and female hoverflies and transferred to 3.5% glutaraldehyde (prepared with a phosphate buffer solution at pH 7.4) containing 0.6% (v/v) TWEEN-20 and 0.09% (w/v) NaCl. Antennae were prefixed for 2 days at 4°C and then washed ten times in phosphate buffer (0.1 mol/L, pH 7.4) for 15 min each time. Next, postfixation antennae were incubated at room temperature for 2 h in a solution of 1% osmium tetroxide mixed with a phosphate buffer solution at a pH of 7.4. After four washes with phosphate buffer, specimens were dehydrated in ethanol solutions (30, 50, 60, 70, 80, and 90%) for 15 min each, followed by two washes in 100% ethanol for 20 min. The specimens were rinsed again and dehydrated with 100% acetone six or seven times for 10 min each, then embedded in a mixture of anhydrous acetone and Spurr’s resin (a ratio of 3:1 for 4 h, 1:1 overnight, 1:3 for 8 h, and in pure resin for 12 h), and polymerized for 72 h at 60°C. Sections (50–80 nm thick) were cut with a LEICA EM UC6 ultramicrotome, transferred onto a copper grid, and stained in saturated uranyl acetate and 1% lead citrate for 10 min each, followed by air-drying. Finally, specimens were observed using a HITACHI H-7,500 (Hitachi, Tokyo, Japan) transmission electron microscope at the Electronic Microscopy Centre of the Institute of Food Science and Technology, CAAS (Beijing, China) operated at 80 kV. The terminology of the antennal segments follows *D. melanogaster* (Shanbhag et al., 1999), whereas sensilla types were classified according to the previous references (Lewis, 1971; Honda et al., 1983; Smallegange et al., 2008; Oh et al., 2019).

### Single Sensillum Recording

A single hoverfly was restrained in a 200 μl plastic pipette tip with the narrow end cutoff. The hoverfly was gently pushed until its head protruded from the cut end and fixed to the rim of the pipette tip with dental wax. Then, one of the exposed antennae was stuck to a coverslip with a double-face adhesive tape. Before recording, tungsten wire electrodes were electrolytically sharpened by repeatedly immersing the tip into 40% KNO_2_ solution. The reference electrode was placed in the hoverfly eye, and the recording electrode was gently inserted into the base of the basiconic sensillum. The recordings were performed under a LEICA Z16 APO microscope at × 920 magnification. A continuous stream of purified and humidified air was directed onto the antenna through a 14 cm-long steel tube controller (Syntech, Hilversum, the Netherlands). Tested odorants were injected into the air stream by a Syntech Stimulus controller (CS-55 model, Syntech), which generated 300 ms air pulses with an airflow of 20 ml/s delivered through a Pasteur pipette. Compensating airflow was provided to keep a constant air stream, but the compensatory flow was switched off during stimulation. Signals of the action potentials were amplified 10 × by a preamplifier (IDAC-4 USB System, Syntech, Kirchzarten, Germany) and then sent to a computer *via* an analog-to-digital converter. Software package Autospike 32 (Syntech) was used to amplify, digitize, and visualize the action potentials. The number of ORNs housed in a single sensillum could be deduced based on the differences in their spike amplitudes. Responses were calculated by the difference between the spike number counted 1 s before and 1 s after delivery of the stimulus. The data were shown as mean ± SEM. GraphPad PRISM version 6.0 software (San Diego, California, United States) was used to make the graphics.

### Odor Stimulation

Ten representative floral scent compounds (98–99% minimum purity) were purchased from Sigma-Aldrich Co. (St. Louis, MO, United States) and used for electrophysiological recordings (Table 1). All odorants were diluted to a final concentration of 100 μg/μl in dimethyl sulfoxide (DMSO). For stimulus delivery, 10 μl of each solution was dripped on a filter paper strip (0.5 × 4 cm) inserted in a Pasteur pipette (15 cm long). DMSO alone was tested as a negative control.

**TABLE 1:** Ten floral scent compounds used for electrophysiological recordings.

Stimulus compounds	CAS number	Purity (%)	Company	References
**Aromatics**				
p-anisaldehyde	123-11-5	98	Sigma	Zito et al., 2019
4-methoxybenzyl alcohol	105-13-5	98	Sigma	Ervik et al., 1999
Methyl eugenol	93-15-2	98	Sigma	Solís-Montero et al., 2018
Eugenol	97-53-0	99	Sigma	Solís-Montero et al., 2018
2-phenylethanol	60-12-8	99	Sigma	Braunschmid et al., 2017
Methyl salicylate	119-36-8	99	Sigma	Solís-Montero et al., 2018
**Terpenoids**				
Linalool	78-70-6	97	Sigma	Zito et al., 2019
Geranyl acetate	105-87-3	97	Sigma	Braunschmid et al., 2017
*Trans*-β-farnesene	18794-84-8	90	Sigma	Braunschmid et al., 2017
**Heterocyclic derivatives**				
Indole	120-72-9	99	Sigma	Braunschmid et al., 2017

## Results

### Gross Morphology of Antennae, Sensilla Types, and Distribution

**FIGURE 1:**
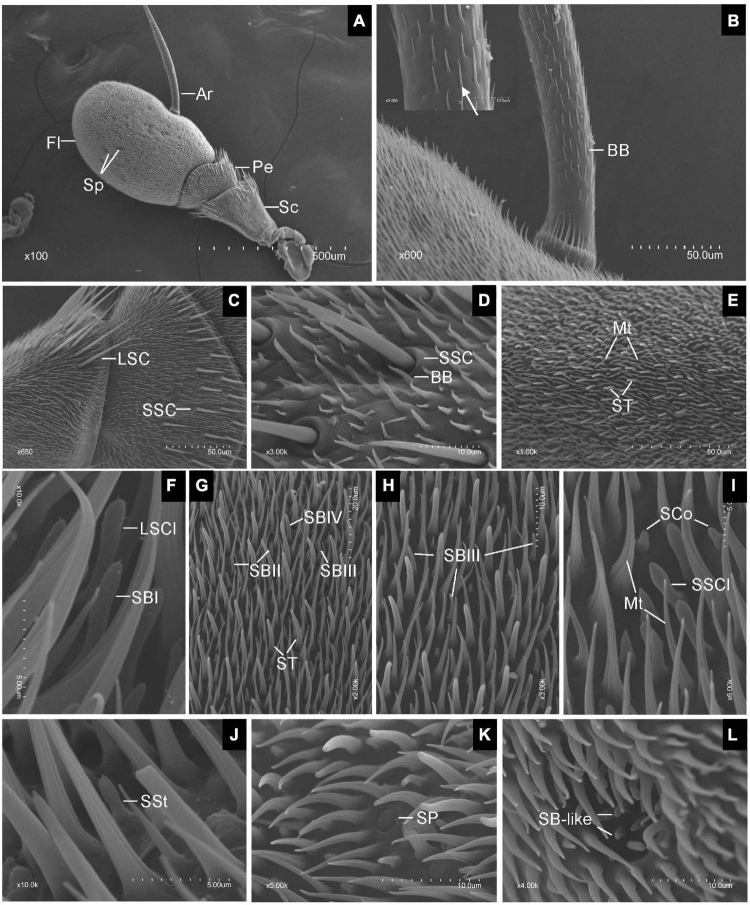
Scanning electron micrographs of *Eupeodes corollae* antenna. **(A)** General view of the antenna from *E. corollae*, showing the scape, the pedicel, the flagellum, the arista, and the sensory pit. **(B)** Böhm bristles spread over the entire arista. Inlay: enlarged view of Böhm bristles (arrows). **(C)** Long sensilla chaetica and short sensilla chaetica in the distal region of the scape and pedicel. **(D)** Short sensilla chaetica with longitudinal furrows interspersed by Böhm bristles in the distal region of the pedicel. **(E)** Sensilla trichodea are long and tower over the layer of microtrichiae on the surface of the flagellum. **(F)** Large sensilla clavate with distal swelling and subtype of SBI with round tip on the surface of the flagellum. **(G)** Funicular subtype of SBII, SBIII, and SBIV. **(H)** Funicular subtype of SBIII with a relatively slender curved tip. **(I)** Small sensilla clavate and sensilla coeloconica. **(J)** Sensilla styloconica with closely apposed finger-like ridges along with the peg. **(K)** Sensilla placodea are like a button with smooth surface. **(L)** Sensory pit with basiconic-like sensilla. Sc, scape; Pe, pedicel; Fl, flagellum; Ar, arista; Sp, sensory pit; BB, Böhm bristle; LSC, long sensilla chaetica; SSC, short sensilla chaetica; ST, sensilla trichodea; Mt, microtrichiae; LSCl, large sensilla clavate; SBI, sensilla basiconica I; SBII, sensilla basiconica II; SBIII, sensilla basiconica III; SBIV, sensilla basiconica IV; SSCl, small sensilla clavate; SCo, sensilla coeloconica; SSt, sensilla styloconica; SP, sensilla placodea. Scale bars in **(F,I,J)** 5 μm; **(D,H,K,L)** 10 μm; **(G)** 20 μm; **(B,C,E)** 50 μm; **(A)**, 500 μm.

Scanning electron microscopy observation revealed that the antennae of both sexes of *E. corollae* were composed of three segments, scape (Sc), pedicel (Pe), and flagellum (Fl). The Fl had a typical structure similar to that of the *Drosophila* counterpart and bore a long arista (Ar) arising from the proximal dorsal ridge (Figure 1A). Short and long hairs and numerous sensilla were present on the aristate Fl. No clear differences in the gross morphology of the antennae were found between male and female *E. corollae*. In total, ten morphologically distinct types of sensilla were observed externally on the antennae of female and male *E. corollae* and included Böhm bristles (BB), sensilla chaetica (SC), microtrichiae (Mt), sensilla trichodea (ST), sensilla basiconica (SB), sensilla clavate (SCl), sensilla coeloconica (SCo), sensilla styloconica (SSt), sensilla placodea (SP), and sensory pit.

Generally, BB spread over the surface of the antennal Sc and Pe segments, and the entire Ar. They displayed a small needle-like structure with a pointed tip, a smooth surface, and stood at an acute angle to the antennal surface. BB did not contain pores, longitudinal grooves, or a flexible socket (Figure 1B). SC was the longest sensilla found on the antennae of both sexes of *E. corollae*. Based on sensillum size, the SC was classified into two subtypes: long SC (LSC) and short SC (SSC) (Figure 1C). LSC was observed in the distal region of the Sc and Pe interspersed by numerous bristles, whereas SSC was only found on the Pe. These sensilla were characterized by thorn-shaped straight hairs incised with longitudinally arranged furrows. The base of the sensillum was inserted into a round socket that stands above the surface of the antenna. The tips of the stout sensilla were apiculiform and did not have pores in the wall (Figure 1D).

Microtrichiae densely covered the entire flagellum and were interspersed around some types of sensilla with no differences between sexes. These sensilla were slender, curved, hair-like structures with longitudinal grooves from base to tip. There was no distinct cuticular socket at the base (Figure 1E). ST was numerous and widely distributed on the surface of the antennal Fl, with the longest one towering over the layer of Mt. ST were present around the distal part of the Fl with fewer toward proximal and ventral regions. Their base arose from a small cuticular pit and tapered to a fine tip, showing a long, hair shape (Figure 1E). SB covered every region of the surface of the Fl. According to their external characteristics, SB can be classified into four subtypes. The subtype of SBI was finger shaped with a rounded tip and slightly curved under the tip (Figure 1F). The subtype of sensilla basiconica II (SBII) was the shortest, in the shape of sturdy pegs tapering from the base to the apex (Figure 1G). The subtype of sensilla basiconica III (SBIII) was relatively slender and somewhat curved (Figures 1G,H). The subtype of sensilla basiconica IV (SBIV) was similar to SBII in shape but much longer (Figure 1G).

Sensilla clavate had a club-like shape and were less widespread on the surface of the flagellum. SCl occurred in two subtypes, large (LSCl) and small (SSCl). SSCl was shorter and stouter than the LSCl. They were morphologically similar to the SB, but have a distal swelling (Figures 1F,I). SCo was quite short and generally located near the proximal region of the Fl on its dorsal and ventral sides. These sensilla arise from a shallow socket. The base of the hair shaft was slightly inflated and gradually tapered to a conical tip. The top two-thirds of SCo had deep longitudinal surface grooves, while the bottom one-third of the sensilla base was smooth, with no grooves or pores (Figure 1I). SSt were generally located on the anterior and posterior surface of the flagellum irregularly at low density. These sensilla were characterized by longitudinally grooved pegs protruding from the antennal surface that tapered into a rounded tip. The base of the SSt was smooth, with no grooves or pores. The top half of the SSt had about nine closely apposed finger-like ridges along with the peg. Some of them terminated below the tip so that the tip may have eight fingers or less (Figure 1J).

Sensilla placodea were scattered irregularly on the surface of the flagellum, like a small button. The shape of SP was like a smooth plate with a small ring around it. There was no pore or distinct socket on the surface of SP (Figure 1K). Only one type of SP was identified in each Fl of both male and female *E. corollae*. These pits have a roundish opening and are surrounded by Mt near the central region of the Fl on its dorsal and ventral sides. The SP appears as hemispherical invaginations and their inner surface was covered by basiconic-like sensilla (Figure 1L). These sensilla resemble the SB on the external surface of the antennal Fl but were slightly curved and smaller than SBII.

### Fine Structure of Antennal Sensilla

#### Sensilla Trichodea

**FIGURE 2:**
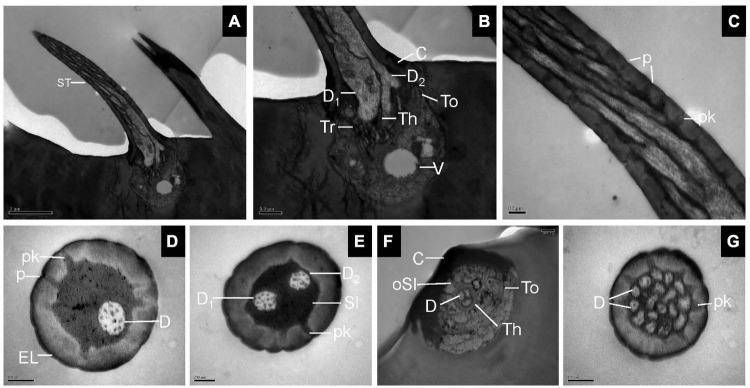
Sensilla trichodea on the antennal surface of *E. corollae* in longitudinal and cross-sections. **(A)** Longitudinal section through the entire ST showing a long, hair-like shape. **(B)** Longitudinal section through the base of the ST, with two outer dendritic segments surrounded by thecogen, trichogen, and tormogen cells. The outer sensillum-lymph cavity contains a clear vesicle, possibly representing extracted lipid droplets. **(C)** Longitudinal section through the hair shaft of the ST at about half-length. The ST were thick-walled and perforated by pores widening into a relatively small pore kettle. **(D–F)** Cross-sections through the basal part of the ST, showing one, two, or three dendrites in the lymph of the sensillum lumen. **(G)** Cross-section through the tip of the ST, showing up to 17 branched dendrites. D, dendrite; Th, thecogen cell; Tr, trichogen cell; To, tormogen cell; V, vesicle; p, pore; pk, pore kettle; EL, epicuticular layers; C, cuticle; Sl, sensillum lymph; oSl, outer sensillum lymph. Scale bars in **(C–G)** 0.2 μm; **(B)** 0.5 μm; and **(A)** 2 μm.

Transmission electron microscopy observations showed that ST was thick-walled and perforated by numerous pores. The wall pores widen into a relatively small pore kettle, which is connected to the sensillum lumen. Two dendrites at the basal region of the ST were bordered by thecogen, trichogen, and tormogen cells (Figures 2A–C). Cross-sections at the base of the sensilla showed one, two, or three dendrites in the lymph of the sensillum lumen (Figures 2D–F). Up to 17 cross-sections of dendrites were seen at the tip of the sensilla (Figure 2G).

#### Sensilla Basiconica

**FIGURE 3:**
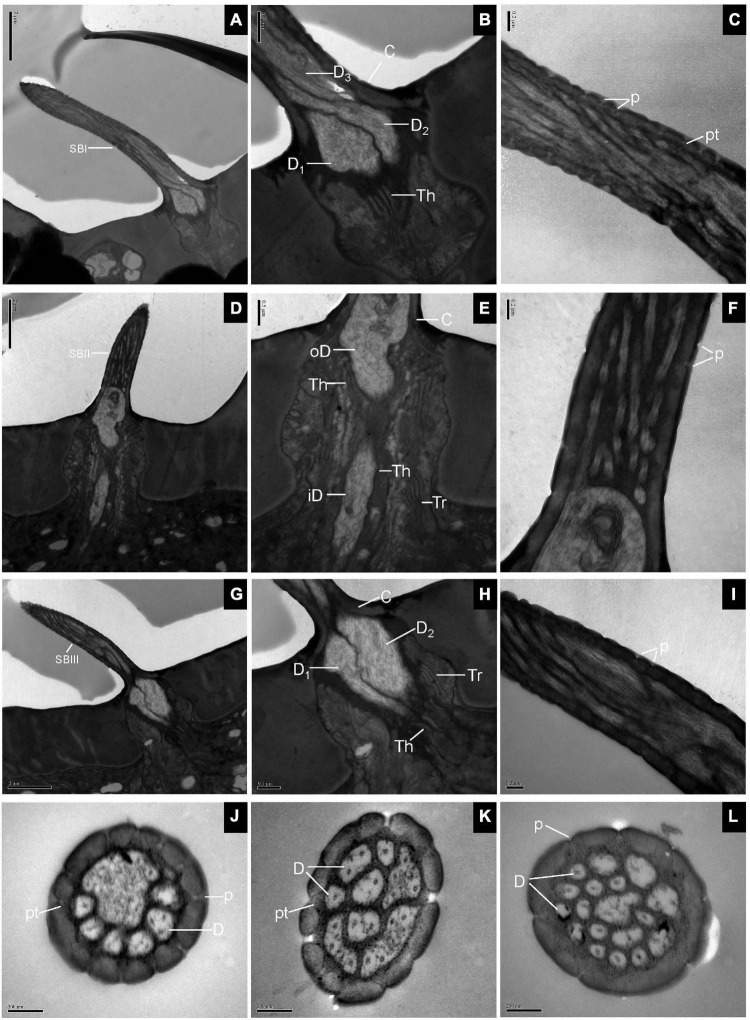
Sensilla basiconica on the antennal surface of *E. corollae* in longitudinal and cross-sections. **(A–C)** Longitudinal section of subtype of SBI. Close to the base of SBI, three dendrites have different diameters and start branching at different levels. The outer dendritic segments are surrounded by thecogen cells. Many pores with pore tubules perforate the thin wall. **(D–F)** Longitudinal section of subtype of SBII. Close to the sensillar base, an outer dendritic segment sends out many dendrites, like a brush. Thecogen and trichogen cells tightly surround the inner dendritic segments at the base of SBII. The hair shaft is pierced by numerous pores. **(G–I)** Longitudinal section of subtype of SBIII. The hair shaft is a little longer and slenderer than SBII. The two dendrites are surrounded by thecogen and trichogen cells, and branch at different levels. The thin cuticular wall is perforated by narrow pore openings. **(J–L)** Cross-sections through various subtypes of sensilla basiconica on the antenna of *E. corollae*. The cuticular wall of sensilla basiconica is relatively thin and has numerous nanoscale wall pores. The outer pore widens into a visible pore tubule and contacts the sensillum lymph in the sensilla lumen. The shape and number of dendritic branches appeared to be different in different subtypes of sensilla basiconica. iD, inner dendritic segment; oD, outer dendritic segment; pt, pore tubule. Other abbreviations followed Figures 1, 2. Scale bars in **(C,F,I–L)** 0.2 μm; **(B,E,H)** 0.5 μm; **(A,D,G)** 2 μm.

Longitudinal sections of SB showed a thin, homogeneous cuticle through the hair shaft that was pierced by numerous, visible pores. Sections through the base of SBI showed three dendrites branching profusely when entering the sensillum lumen (Figures 3A,B). The wall pores were distributed all around the sensilla, and typical pore tubules extended into the lumen (Figure 3C). Near the SBII base, an outer dendritic segment splits into abundant dendritic branches. This branching was restricted to a small region, resulting in a brush-like structure. The inner dendritic segment was tightly surrounded by thecogen and trichogen cells (Figures 3D,E). There were many fewer wall pores compared with SBI (Figure 3F). At the base of SBIII, the thecogen cells closely contacted two dendrites that extended either in longitudinal or in angled directions in the sensillum lumen (Figures 3G,H). The pore density gradually increased toward the sensilla tip (Figure 3I). Cross-sections through various subtypes of SB showed numerous nanoscale pores and pore tubules that perforated the comparatively thin cuticular wall. The outer dendritic segments in different subtypes of SB may branch at somewhat different levels (Figures 3J–L).

#### Sensilla Clavate

**FIGURE 4:**
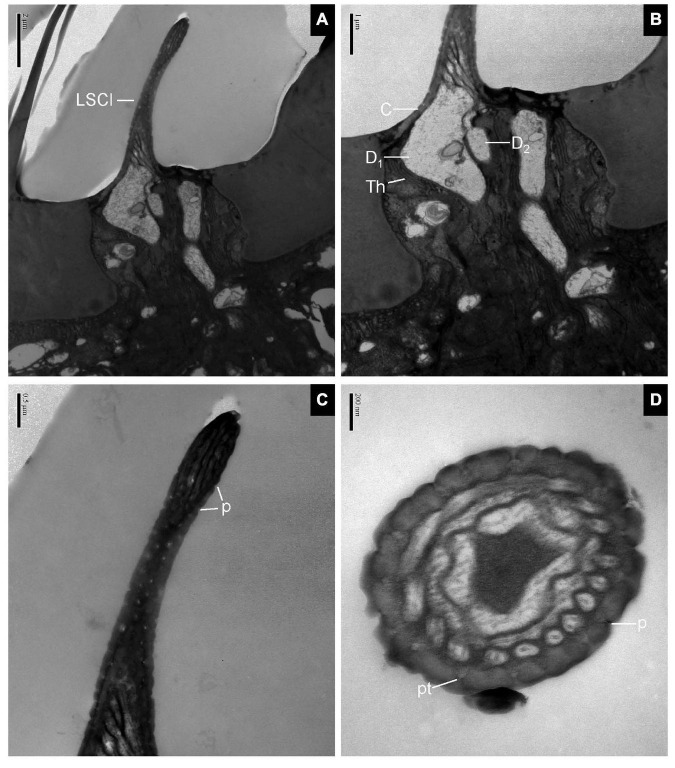
Large sensilla clavate in longitudinal and cross-sections on the antennal surface of *E. corollae*. **(A–C)** Large sensilla clavate in longitudinal section. The distal swelling of the sensilla is conspicuous, like a wooden club. The two dendrites varying in sizes are split into many dendritic branches in the sensilla lumen. The pore density is somewhat higher on the relatively thin cuticular wall. **(D)** Large sensilla clavate in cross-section. The lumen of the sensilla contains highly lamellated dendrites. Abbreviations as in Figures 1, 2. Scale bars in **(D)** 0.2 μm; **(C)** 0.5 μm; **(B)** 1 μm; and **(A)** 2 μm.

The longitudinal sections of LSC exhibited two dendrites varying in size that sends out numerous branches in the sensillum lumen (Figures 4A,B). The sensilla wall had many pores with pore tubules and their densities were comparable to those of the SB. The pore density at the proximal base gradually decreased (Figure 4C). Dendrites in the sensilla lumen were highly lamellated different from those of the SB (Figure 4D). No ultrastructure of SSC was observed in this experiment.

#### Sensilla Coeloconica, Sensilla Chaetica, Microtrichiae, and Böhm Bristles

**FIGURE 5:**
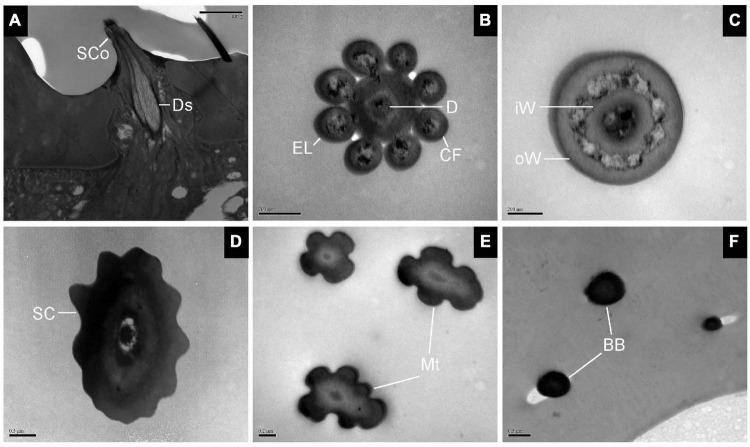
Transmission electron micrographs of sensilla coeloconica, sensilla chaetica, microtrichiae, and Böhm bristles on the antennal surface of *E. corollae*. **(A–C)** TEM micrographs of sensilla coeloconica. **(A)** The longitudinal section through the peg shows a smooth shaft at the base and a deep groove at the distal end. The internal lumen is filled with two unbranched dendrites in a visible dendrite sheath. **(B)** Cross-section through the tip of sensilla coeloconica shows eight cuticular fingers surrounding the central lumen, where only one dendrite is visible. **(C)** Cross-section through the base of the sensilla coeloconica, showing the double-walled structure of the sensillum. **(D)** Cross-section through sensilla chaetica, showing a thick non-porous cuticular wall. No neuronal dendrite is present, but there is a narrow tubular space near the center lumen. **(E)** Cross-section through the microtrichiae, showing no dendrite in the sensilla central lumen. **(F)** Cross-section through the cuticular peg of a Böhm bristle, showing a thick aporous cuticular wall, and no sensory neurons. Ds, dendrite sheath; CF, cuticular finger; EL, epicuticular layers; iW, inner wall; oW, outer wall. Other abbreviations as in Figures 1, 2. Scale bars in **(B,C,E)** 0.2 μm; **(D,F)** 0.5 μm; **(A)** 2 μm.

The longitudinal section of SCo showed two outer dendritic segments tightly surrounded by a dendrite sheath and which extended unbranched into the sensilla lumen (Figure 5A). The distal cross-section of the sensillum had a rosette-shaped structure. The inner walls appeared to be fused. The outer walls of neighboring cuticular fingers are separated by grooves (Figure 5B). Therefore, pores may be present between the grooves. Basal cross-section exhibited the double-walled structure of the SCo. The lumen of the inner cuticle contained dendrite segments surrounded by electron-dense sensillum lymph. The space between the inner and outer cuticular walls was full of filament-like electron-dense structures (Figure 5C). The ultrastructure of the SC showed an obvious thick and aporous cuticular wall. There was no neuronal dendrite but narrow tubular space was observed in the sensilla lumen. The cuticular wall was bordered by visible ridges due to the external furrows (Figure 5D). The cross-section of Mt showed that the sensilla were not innervated (Figure 5E). The ultrastructure of BB showed that the cuticle was much thicker than that of the olfactory sensilla, and the neuronal dendrites were absent (Figure 5F).

### Responses of Neurons Housed in SBI to Floral Scent Compounds

**FIGURE 6:**
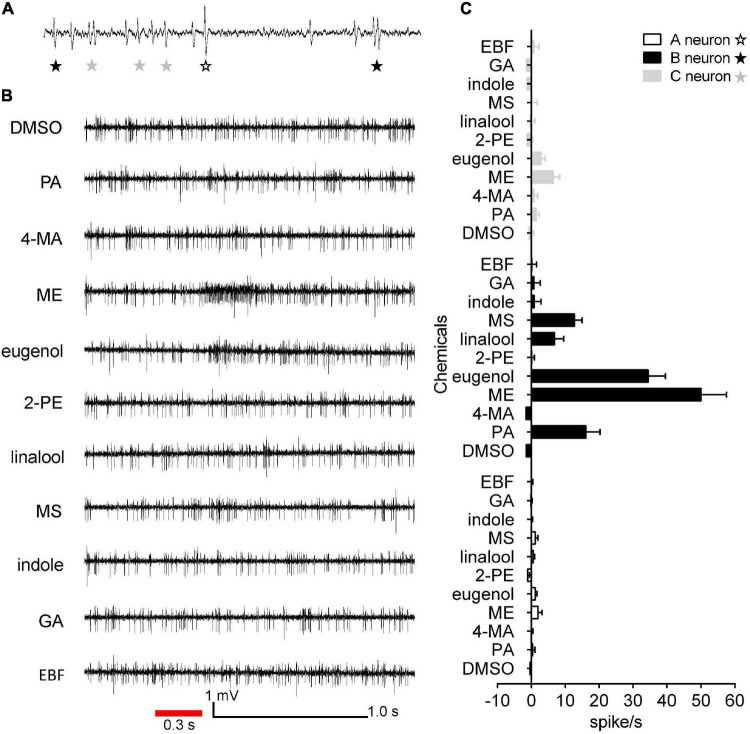
Single sensillum recordings from subtype of SBI of female and male *E. corollae* responding to ten floral scent compounds. **(A)** Spontaneous activity (1 s) of subtype of SBI. Individual action potentials (spikes) were labeled A, B, or C according to their spike amplitude. **(B)** Representative traces of neurons in SBI stimulated by *p*-anisaldehyde, 4-methoxybenzyl alcohol, methyl eugenol, eugenol, 2-phenylethanol, linalool, methyl salicylate, indole, geranyl acetate, and (*E*)-β-farnesene; stimulus 1 mg (100 μg/μl). The red line represents the 0.3 s odorant stimulation. **(C)** Mean SSR responses of A, B, and C neurons from SBI stimulated by ten representative floral scent compounds. Neuron B in SBI was strongly activated by two odorants, eugenol, and methyl eugenol. Neurons A and C, by contrast, had no responses to any of the tested odorants. Responses were calculated by the difference between the spike number counted 1 s before and 1 s after delivery of the stimulus. *n* = 8. DMSO, dimethyl sulfoxide; PA, *p*-anisaldehyde; 4-MA, 4-methoxybenzyl alcohol; ME, methyl eugenol; 2-PE, 2-phenylethanol; MS, methyl salicylate; GA, geranyl acetate; EBF, (*E*)-β-farnesene.

To evaluate the olfactory neuron responses of sensilla of *E. corollae* to floral volatiles, we performed SSR in SB and ST from antennae in both sexes using ten representative floral scent compounds (Table 1). Fortunately, the neurons housed in SBI were found activated by these chemicals. Spontaneous activity of neurons housed in the SBI revealed that three neurons were distinguishable as A, B, and C, based on the spike amplitudes (Figure 6A). The result further confirmed that three dendrites were observed in the SBI. Neuron B in the SBI was strongly activated by methyl eugenol and eugenol at doses of 1 mg. The strongest response to methyl eugenol was 50 ± 7.57 spikes/s. *p*-Anisaldehyde, linalool, and methyl salicylate also elicited moderate responses of neuron B. By contrast, neurons A and C did not respond to any of these odorants (Figures 6B,C).

## Discussion

Syrphids represent a diverse and economically important family in Diptera, comprising about 6,697 described species in 283 genera, with most species distributed in the Neotropical, Nearctic, and Palaeotropical regions.^1^ Despite their significant roles as pollinators and predators in chemoreception (Dunn et al., 2020), there are few studies regarding the ultrastructural organization of their sensory organs (Henderson and Wellington, 1982; Skevington and Dang, 2002; Larson et al., 2012; Jia et al., 2019). In this study, based on SEM observations, we identified ten morphologically distinct types of sensilla on antennae of female and male *E. corollae*, including Böhm bristles, sensilla chaetica, microtrichiae, sensilla trichodea, sensilla basiconica, sensilla clavate, sensilla coeloconica, sensilla styloconica, sensilla placodea, and sensory pit. Böhm bristles and sensilla chaetica were distributed on the scape and pedicel of *E. corollae* antenna, while other innervated sensilla were densely distributed on the flagellum, indicating the predominant sensory function of this segment.

The morphological traits and different types of sensilla are similar to those of other Dipteran Cyclorrhapha species (Supplementary Table 1). But two traits in *E. corollae* make the antennae different from those of other Diptera species. First, the sensilla styloconica was short and had grooved pegs on the flagellum of hoverflies. However, as in *Hydrotaea irritans* (Muscidae) (Been et al., 1988), *Toxotrypana curvicauda* (Arzuffi et al., 2008), and *Anastrepha fraterculus* (Tephritidae) (Bisotto-De-Oliveira et al., 2011), they are mainly present on the antennal pedicel among most calyptrate families (Supplementary Table 1), including Fanniidae, Anthomyiidae, Muscidae, Sarcophagidae, and Calliphoridae. Furthermore, these SSt have two primary morphologic types. These sensilla with longitudinal grooves along with the pegs are gradually tapered in Tephritidae and Syrphidae (Arzuffi et al., 2008; Bisotto-De-Oliveira et al., 2011). Throughout evolutionary history, this state has changed in most calyptrate families: the pegs have been transformed into setae that are bulbous at the base, and acute or obtuse at the distal end (Sukontason et al., 2004; Hore et al., 2017). Therefore, the distribution and morphology of SSt offer important information about the evolutionary history of Cyclorrhapha taxa, suggesting valuable potential in the phylogenetic signal of this structure. Second, the presence of sensilla placodea, already described on Hymenopteran (Xi et al., 2011; Silva et al., 2016) and Coleopteran antennae (Liu et al., 2012), was shown in a Dipteran species for the first time. Further electrophysiological studies are needed to verify the actual functions of those sensilla in *E. corollae*.

In addition, ultrastructural organization of sensilla trichodea, sensilla basiconica, large sensilla clavate, sensilla coeloconica, sensilla chaetica, Böhm bristles, and microtrichiae were obtained under TEM, providing fine morphological evidence of their possible sensory functions. The common morphological feature for olfactory sensilla is the multiple nanoscale wall pores on the surface of the hair shaft (Steinbrecht, 1997; Shanbhag et al., 1999). Our study showed that sensilla trichodea and all subtypes of sensilla basiconica from *E. corollae* antennae contain multiple nanoscale wall pores, suggesting that their major function is olfaction. Additionally, some basiconic-like sensilla are detected in the sensory pit near the central region of the flagellum, which are consistent with most previous morphological studies in *Delia radicum*, *D. floralis*, *D. antiqua*, *D. platura*, *Hypoderma bovis*, *Protophormia terraenovae*, *Fannia scalaris*, and *F. canicularis* (Ross, 1992; Hunter and Adserballe, 1996; Setzu et al., 2011; Zhang et al., 2013a). The convergence of sensilla in pits could expose a larger surface to receive odorants efficiently, increase sensitivity, and also protect the delicate sensilla from mechanical deformation (Ross, 1992; Hunter and Adserballe, 1996; de Bruyne et al., 2001; Zhang et al., 2012a,b, 2013a,b).

Moreover, we classified the LSC as an independent sensilla type due to the swelling in the distal region and the highly lamellated dendrites even though it has multiple walls pores on the surface like SB. LSCs have been observed and defined in *Hylemya antiqua* (Honda et al., 1983) and *Musca domestica* (Smallegange et al., 2008). The LSCs are widespread on the antennal flagellum, implying that they may have an important chemosensory function in detecting various chemical compounds related to certain behavior in *E. corollae*. An exception is sensilla coeloconica, which has deep longitudinal grooves along with the central peg instead of wall pores, also suggesting an olfactory function. TEM observation in the tobacco hornworm *Manduca sexta* (Shields and Hildebrand, 1999) and *D. melanogaster* (Yao, 2005) indicated that the intergroove region of sensilla coeloconica may be the entry point of odorants. Furthermore, cross-sections of the sensilla chaetica, Böhm bristles, and microtrichiae did not exhibit any wall pores on the cuticular surface nor any sensory cells at their base. The sensilla observed in this study are common to other Diptera families such as Muscidae (Been et al., 1988; Smallegange et al., 2008), Tephritidae (Hu et al., 2010; Liu Y. et al., 2020), Sarcophagidae (Liu et al., 2016; Pezzi et al., 2016), and Drosophilidae (Gao et al., 2019), and are considered to have a mechanosensory function.

Odorant receptor neurons housed in the olfactory sensilla with multiple walls pores on the antennae are the first relay center between external odor stimuli and second-order neurons in the brain where the information is further processed (Wang et al., 2003; Wilson, 2004; de Bruyne and Baker, 2008). ORNs can be divided into distinct functional classes based on their odorant response spectra (de Bruyne et al., 2001; Yao, 2005; Soni et al., 2019). In this study, first, we evaluated the olfactory neuron responses in the subtype of SBI on the antennae of female and male *E. corollae* to ten floral scent compounds by SSR. We identified three ORNs housed within a subtype of SBI based on the different spike amplitudes, corresponding to the numbers of neuronal dendrites observed under TEM (Figure 3B). Our results showed that neuron B in the subtype of SBI is mainly responsible for detecting floral scent compounds, especially aromatic compounds. Similar findings in other species were reported in terms of the response of ORNs in the sensilla basiconica to aromatics. For example, in model insect *D. melanogaster*, sensilla basiconica usually houses two to four neurons, in which ab1D responded with a high degree of specificity to methyl salicylate (de Bruyne et al., 2001). In *Anoplophora glabripennis* (Coleoptera: Cerambycidae), ORNs in blunt-tipped sensilla basiconica were responsive to eugenol and some terpenoids (Wei et al., 2018). Moreover, the action potentials of ORNs lead to the activation of the second-order neurons in the brain to produce behavioral changes (Wei et al., 2018). A recent study showed that an odorant receptor EcorOR25 was narrowly tuned to aromatic compounds, particularly eugenol and methyl eugenol, which can strongly attract *E. corollae* adults of both sexes (Li et al., 2020). However, whether EcorOR25 is expressed in the ORNs of subtype of SBI or other sensilla remains unknown because not all types of SB were recorded due to the dense sensilla and microtrichiae growing on the antenna. Hence, the receptor-to-neuron map of the olfactory sensilla in the peripheral nervous system of the *E. corollae* antenna is required for further studies.

## Conclusion

In conclusion, ten types of sensilla on the antennae of male and female *E. corollae* were identified based on morphology, and the fine structure was characterized using SEM and TEM techniques. Our study also indicates that neuron B in the SBI primarily responds to aromatic compounds. These results provide a basis for functional studies of the ORNs in *E. corollae*. The detailed ultrastructural descriptions of antennal sensilla may provide critical data for taxonomic and phylogenetic studies of Diptera.

## Data Availability Statement

The original contributions presented in the study are included in the article/Supplementary Material, further inquiries can be directed to the corresponding author/s.

## Author Contributions

W-YD, BW, and G-RW designed the experiments, wrote, and revised the manuscript. W-YD and BW performed the experiments and analyzed the data. BW and G-RW contributed reagents and materials. All authors contributed to the article and approved the submitted version.

## Conflict of Interest

The authors declare that the research was conducted in the absence of any commercial or financial relationships that could be construed as a potential conflict of interest.

## Publisher’s Note

All claims expressed in this article are solely those of the authors and do not necessarily represent those of their affiliated organizations, or those of the publisher, the editors and the reviewers. Any product that may be evaluated in this article, or claim that may be made by its manufacturer, is not guaranteed or endorsed by the publisher.

## Footnotes

1
https://doi.org/10.15468/39omei
