# Supplementary material for: Morphological and Ultrastructural Characterization of Antennal Sensilla and the Detection of Floral Scent Volatiles in Eupeodes corollae (Diptera: Syrphidae)
Source: Front Neuroanat. 2021 Dec 16;15:791900. doi: 10.3389/fnana.2021.791900 (PMC8716465; doi:10.3389/fnana.2021.791900)
Supplement: Supplementary file 1 [file Data_Sheet_1.docx]

**Table S1 Morphological and ultrastructural comparison of antennae sensilla from *Eupeodes corollae* and other Diptera species**

| **Species** | **Number of subtypes of ST** | **Number of subtypes of SB** | **Number of subtypes of SCo** | **Number of subtypes of SC** | **Number of subtypes of SCl** | **Number of subtypes of SSt** | **Number of subtypes of SAu** | **Number of sensory pit** | **Type of sensilla in sensory pit** | **Sex dimorphism** | **Reference** |
| --- | --- | --- | --- | --- | --- | --- | --- | --- | --- | --- | --- |
| **Syrphidae** |  |  |  |  |  |  |  |  |  |  |  |
| *Eupeodes corollae* | 1(Fn) | 4(Fn) | 1(Fn) | 1(Sc),  2(Pd) | 1(Fn) | 1(Fn) | ─ | present, number not given | basiconic-like s. | no | this paper |
| *Eupeodes volucris* | 1(Fn) | 2(Fn) | 1(Fn) | ─ | ─ | ─ | ─ | present, number not given | ─ | no | (Henderson and Wellington, 1982) |
| *Metasyrphus venablesi* | 1(Fn) | 2(Fn) | 1(Fn) | ─ | ─ | ─ | ─ | present, number not given | ─ | no | (Henderson and Wellington, 1982) |
| **Fanniidae** |  |  |  |  |  |  |  |  |  |  |  |
| *Fannia scalaris* | 1(Fn) | 2(Fn) | 1(Fn) | ─ | 1(Fn) | ─ | ─ | 1(ds), 1-2(vs),6-10 (lateral region), one sacculus near pits | basiconic-like s. | only male | (Zhang et al., 2013a) |
| *Fannia canicularis* | 1(Fn) | 2(Fn) | 1(Fn) | ─ | 1(Fn) | 1(Pd) | ─ | 1(ds), 1-2(vs),6-10 (lateral region), one sacculus near pits | basiconic-like s. | only male | (Zhang et al., 2013a; Greenberg and Ash, 1972) |
| *Fannia hirticeps* | 1(Fn) | 2(Fn) | 2(Fn) | 1(Sc, Pd) | 1(Fn) | ─ | ─ | 1(vs) | ─ | no | (Wang et al., 2012) |
| **Anthomyiidae** |  |  |  |  |  |  |  |  |  |  |  |
| *Delia radicum* | 1(Fn) | 2(Fn) | 1(Fn) | ─ | 1(Fn) | 1(Pd) | ─ | 3(ds), 1(vs)-female; 4(ds), 1(vs)-male | basiconic II s.(ds); grooved pit s., smooth-walled conical pit s., smooth-walled tapered pit s., striated pit s.(vs) | yes | (Ross, 1992) |
| *Delia floralis* | 1(Fn) | 2(Fn) | 1(Fn) | ─ | 1(Fn) | 1(Pd) | ─ | 3(ds), 1(vs)-female; 4(ds), 2(vs)-male | basiconic II s. (ds); grooved pit s., smooth-walled conical pit s., striated pit s. (vs) | yes | (Ross, 1992) |
| *Delia antiqua* | 1(Fn) | 2(Fn) | 1(Fn) | ─ | 1(Fn) | 1(Pd) | ─ | 8(ds), 1(vs)-female; 8(ds), 1(vs)-male | basiconic II s. (ds); grooved pit s., smooth-walled conical pit s., striated pit s. (vs) | yes | (Ross, 1992) |
| *Delia platura* | 1(Fn) | 2(Fn) | 1(Fn) | ─ | 1(Fn) | 1(Pd) | ─ | 3(ds), 1(vs)-female; 3(ds), 1(vs)-male | basiconic II s. (ds); grooved pit s., smooth-walled conical pit s., striated pit s. (vs) | yes | (Ross, 1992; Wang et al., 2014a) |
| *Hylemya antiqua* | 1(Fn) | 2(Fn) | 1(Fn) | ─ | 1(Fn) | ─ | ─ | One large pit (proximal outer region); 8-10 small pit (proximal inner and ventral region) | grooved s., smooth-walled pit s., striated pit s. (large pit); basiconic s. (small pits) | no | (Honda et al., 1983) |
| **Muscidae** |  |  |  |  |  |  |  |  |  |  |  |
| *Lispe neimongola* | 1(Fn) | 2(Fn) | ─ | 1(Sc),  2(Pd) | 1(Fn) | ─ | ─ | ─ | ─ | no | (Zhang et al., 2013b) |
| *Stomoxys calcitrans* | 3(Fn) | 3(Fn) | 1(Fn) | ─ | 1(Fn) | ─ | ─ | present, number not given | ─ | no | (Tangtrakulwanich et al., 2011) |
| *Hydrotaea irritans* | 1(Fn) | 6(Fn) | ─ | ─ | ─ | 1(Fn) | ─ | present, number not given | thin-walled multiporous s. (ds) | no | (Been et al., 1988) |
| *Hydrotaea chalcogaster* | 1(Sc, Pd) | 2(Fn) | 1(Fn) | ─ | ─ | ─ | ─ | present, number not given | small basiconic s. | no | (Sukontason et al., 2007) |
| *Hydrotaea armipes* | 1(Fn) | 2(Fn) | 1(Fn) | 1(Sc, Pd) | 1(Fn) | ─ | ─ | present, number not given | ─ | no | (Wang et al., 2014b) |
| *Musca autumnalis* | 1(Fn) | 2(Fn) | 1(Fn) | ─ | ─ | 1(Pd) | ─ | 1(ds), 2(vs)-female; 1(ds), 1(vs)-male | pit basiconic s. (vs); pit grooved s., pit filamentous tip s. (ds) | yes | (Greenberg and Ash, 1972; Bay and Pitts, 1976) |
| *Musca domestica* | 1(Sc, Pd) | 2(Fn) | 1(Fn) | ─ | 1(Fn) | 1(Pd) | ─ | 2 Type I (one at the lateral and one at the ventral Fn),1 Type II (ventroproximal Fn)-female, male | grooved s., striated s., conical structures (Type I); clavate s. (Type II) | no | (Sukontason et al., 2004; Smallegange et al., 2008) |
| *Ophyra capensis* | 1(Fn) | 2(Fn) | ─ | 1(Sc),  2(Pd) | ─ | ─ | ─ | ─ | ─ | yes | (Hore et al., 2018) |
| **Tephritidae** |  |  |  |  |  |  |  |  |  |  |  |
| *Bactrocera zonata* | 2(Sc, Pd, Fn) | 1(Sc, Pd), 1(Fn) | 2(Fn) | 1(Sc, Pd) | 1(Fn) | ─ | ─ | ─ | ─ | yes (feed on peach and guava fruit); no (feed on orange) | (Awad et al., 2014; Awad et al., 2015) |
| *Bactrocera depressa* | 1(Fn) | 2(Fn) | 1(Fn) | ─ | ─ | ─ | ─ | ─ | ─ | no | (Oh et al., 2019) |
| *Toxotrypana curvicauda* | 1(Fn) | 1(Fn) | ─ | ─ | 1(Fn) | 1(Fn) | ─ | present, number not given | ─ | yes | (Arzuffi et al., 2008) |
| *Anastrepha fraterculus* | 1(Fn) | 1(Fn) | ─ | 1(Sc, Pd) | 2(Fn) | 1(Fn) | ─ | ─ | ─ | no | (Bisotto-De-Oliveira et al., 2010) |
| *Bactrocera oleae* | 1(Fn) | 1(Fn) | 1(Fn) | ─ | 1(Fn) | ─ | ─ | present, number not given | ─ | no | (Liscia et al., 2013) |
| *Neoceratitis asiatica* | 1(Fn) | 1(Fn) | 1(Fn) | 1(Sc, Pd) | 1(Fn) | ─ | ─ | ─ | ─ | no | (Liu et al., 2020) |
| *Bactrocera tau* | 1(Fn) | 2(Fn) | 1(Fn) | 1(Sc, Pd) | ─ | ─ | ─ | ─ | ─ | no | (Hu et al., 2010) |
| *Bactrocera dorsalis* | 1(Fn) | 2(Fn) | 1(Fn) | 1(Sc, Pd) | ─ | ─ | ─ | ─ | ─ | no | (Hu et al., 2010) |
| *Bactrocera cucurbitae* | 1(Fn) | 2(Fn) | 1(Fn) | 1(Sc, Pd) | ─ | ─ | ─ | ─ | ─ | no | (Hu et al., 2010) |
| *Bactrocera minax* | 1(Fn) | 2(Fn) | 1(Fn) | 1(Sc, Pd) | ─ | ─ | ─ | ─ | ─ | yes | (Hu et al., 2010) |
| *Bactrocera diaphora* | 1(Fn) | 2(Fn) | 1(Fn) | 1(Sc, Pd) | ─ | ─ | ─ | ─ | ─ | no | (Hu et al., 2010) |
| *Bactrocera scutellata* | 1(Fn) | 2(Fn) | 1(Fn) | 1(Sc, Pd) | ─ | ─ | ─ | ─ | ─ | no | (Hu et al., 2010) |
| **Sarcophagidae** |  |  |  |  |  |  |  |  |  |  |  |
| *Sarcophaga tibialis* | 1(post-Pd) | 1(post-Pd) | ─ | 1(Sc),  3(Pd) | 1(post-Pd) | 1(post-Pd) | ─ | 12-21(ds), 13-20(vs)-female; 3-8(ds), 1-5(vs)-male | bottle-shaped s. | yes | (Pezzi et al., 2016) |
| *Parasarcophaga dux* | 1(Sc, Pd) | 2(Fn) | 1(Fn) | ─ | ─ | 1(Pd) | ─ | 8-15(female);  0-6(male) | sensory pegs (female); sensory pegs with bulbous bases (male) | yes | (Sukontason et al., 2004) |
| *Scathophaga stercoraria* | 1(Fn) | 3(Fn) | 1(Fn) | 1(Sc, Pd) | 1(Fn) | 1(Pd) | ─ | present, number not given | basiconic III s. | no | (Liu et al., 2016) |
| **Oestridae** |  |  |  |  |  |  |  |  |  |  |  |
| *Rhinoestrus purpureus* | ─ | 2(Fn) | 1(Fn) | ─ | 1(Fn) | ─ | ─ | 29(ds), 8(vs)-female;23(ds), 5(vs)-male | basiconic I and II s., clavate s., coeloconic s. | yes | (Liu et al., 2015) |
| *Hypoderma lineatum* | 1(Fn) | 2(Fn) | 1(Fn) | ─ | 1(Fn) | ─ | ─ | 31(ds), 25(vs)-female;10(ds), 10(vs)-male | trichoid s., basiconic I and II s., clavate s., coeloconic s. | yes | (Li et al., 2015) |
| *Gasterophilus nigricornis* | 1(Fn) | 3(Fn) | ─ | ─ | 1(Fn) | ─ | 1(Fn) | 60-78 pits in total;34(ds);40(vs), only a few appear on the dorsolateral margin | auriculate s. | only male | (Zhang et al., 2012) |
| *Gasterophilus haemorrhoidalis* | 1(Fn) | 2(Fn) | 2(Fn) | ─ | 1(Fn) | ─ | 1(Fn) | 26(ds), 28(vs)-female;56(ds), 43(vs)-male | ─ | yes | (Zhang et al., 2016) |
| *Gasterophilus intestinalis* | 1(Fn) | 2(Fn) | 2(Fn) | ─ | 1(Fn) | ─ | 1(Fn) | 58(ds), 24(vs)-female;25(ds), 29(vs)-male | ─ | yes | (Zhang et al., 2016) |
| *Gasterophilus nasalis* | 1(Fn) | 2(Fn) | 2(Fn) | ─ | 1(Fn) | ─ | 1(Fn) | 34(ds), 23(vs)-female;23(ds), 12(vs)-male | ─ | yes | (Zhang et al., 2016) |
| *Gasterophilus pecorum* | 1(Fn) | 2(Fn) | 3(Fn) | ─ | 1(Fn) | ─ | ─ | ─ | ─ | yes | (Zhang et al., 2016) |
| **Calliphoridae** |  |  |  |  |  |  |  |  |  |  |  |
| *Protophormia terraenovae* | 1(Fn) | 2(Fn) | ─ | ─ | 1(Fn) | ─ | 1(Fn) | Median area (13.6±0.8-female; 12.1±0.7-male); lateral area (2.4±0.3-female; 2.2±0.3-male) | basiconic-like pit s. | yes | (Setzu et al., 2011) |
| *Chrysomya megacephala* | 1(Sc, Pd) | 2(Fn) | 1(Fn) | ─ | ─ | 1(Pd) | ─ | present, number not given | ─ | no | (Sukontason et al., 2004) |
| *Chrysomya rufifacies* | 1(Sc, Pd) | 2(Fn) | 1(Fn) | ─ | ─ | 1(Pd) | ─ | present, number not given | ─ | no | (Sukontason et al., 2004) |
| *Chrysomya nigripes* | 1(Sc, Pd) | 2(Fn) | 1(Fn) | ─ | ─ | 1(Pd) | ─ | present, number not given | ─ | no | (Sukontason et al., 2004) |
| *Lucilia cuprina* | 1(Sc) 2(Pd) | 2(Fn) | 1(Fn) | ─ | ─ | 1(Pd) | ─ | present, number not given | ─ | no | (Sukontason et al., 2004; Hassan et al., 2013) |
| *Cochliomyia honimivorax* | 2(Pd),  1(Fn) | 3(Fn) | 5(Fn) | ─ | ─ | ─ | ─ | ─ | ─ | yes | (de Fernandes et al., 2004) |
| *Hemipyrellia ligurriens* | 1(Fn) | 1(Fn) | 1(Fn) | 1(Sc), 2(Pd) | ─ | 1(Pd) | ─ | ─ | ─ | yes | (Hore et al., 2017) |
| *Triceratopyga calliphoroides* | 1(Fn) | 3(Fn) | 3(Fn) | ─ | 1(Fn) | ─ | ─ | 5-17 sensory pit (at the junction of dorsolateral margin and posteroventral surface); one sacculus (ds) | basiconic IV s., coeloconic-like s. | no | (Zhang et al., 2014) |
| *Lucilia sericata* | 1(Fn) | 2(Fn) | 2(Fn) | ─ | ─ | 1(Pd) | ─ | present two types, number not given | basiconic or basiconic-like s. (Type I); coeloconic-like s. (Type II) | no | (Zhang et al., 2013c) |
| **Drosophilidae** |  |  |  |  |  |  |  |  |  |  |  |
| *Drosophila melanogaster* | 2(Fn) | 3(Fn) | 2(Fn) | 1(Sc, Pd) | 1(Fn) | ─ | ─ | sacculus (dorso-ventral) with three chambers | no-pore basiconic s., no-pore coeloconic s., grooved s. | yes | (Shanbhag et al., 1995; Shanbhag et al., 1999; Gao et al., 2019) |
| *Drosophila suzukii* | 2(Fn) | 3(Fn) | 1(Fn) | 1(Sc, Pd) | 1(Fn) | ─ | ─ | ─ | ─ | no | (Gao et al., 2019; Jeong et al., 2020) |
| *Drosophila immigrans* | 2(Fn) | 1(Fn) | 2(Fn) | 1(Sc, Pd) | 1(Fn) | ─ | ─ | ─ | ─ | no | (Gao et al., 2019) |
| *Drosophila hydei* | 2(Fn) | 1(Fn) | ─ | 1(Sc, Pd) | 1(Fn) | ─ | ─ | ─ | ─ | no | (Gao et al., 2019) |
| **Hippoboscidae** |  |  |  |  |  |  |  |  |  |  |  |
| *Melophagus ovinus* | ─ | 1(Fn) | 1(Fn) | ─ | ─ | ─ | ─ | ─ | ─ | yes | (Zhang et al., 2015) |
| *Hippobosca equina* | ─ | 1(Fn) | 1(Fn) | ─ | ─ | ─ | ─ | ─ | ─ | yes | (Zhang et al., 2015) |
| *Hippobosca longipennis* | ─ | 2(Fn-female); 1(Fn-male) | 1(Fn) | ─ | ─ | ─ | ─ | ─ | ─ | yes | (Zhang et al., 2015) |
| **Glossinidae** |  |  |  |  |  |  |  |  |  |  |  |
| *Glossina palpalis* | ─ | 2(Fn) | ─ | 2(Sc, Pd) | ─ | ─ | ─ | 3 or 4 (ds), 3 or 4(vs)-female;1 (ds), 1 (vs)-male | basiconic II s. | yes | (Isaac et al., 2015) |
| *Glossina tachinoides* | 1(Sc) | 2(Fn) | ─ | 2(Sc, Pd) | ─ | ─ | ─ | 1 or 2 (female) | basiconic II s. | yes | (Isaac et al., 2015) |

Note: The terms from the original paper are used in the table. ST, sensilla trichodea; SB, sensilla basiconica, SCo, sensilla coeloconica, SC, sensilla chaetica, SCl, sensilla clavate, SSt, sensilla styloconica, SAu, sensilla auricillica; Sc, scape; Pd, pedicel; Fn, flagellum; ds, dorsal surface; vs, ventral surface; s., sensilla; “─” represent no data is shown in original paper.

**References**

Arzuffi, R., Robledo, N., and Valdez, J. (2008). Antennal sensilla of *Toxotrypana curvicauda* (Diptera: Tephritidae). *Fla. Entomol*. 91, 669-673. doi: 10.1653/0015-4040-91.4.669

Awad, A. A., Ali, N. A., and Mohamed, H. O. (2014). Ultrastructure of the antennal sensillae of male and female peach fruit fly, *Bactrocera zonata*. *J. Insect Sci*. 14, 45. doi: 10.1093/jis/14.1.45

Awad, A. A., Mohamed, H. O., and Ali, N. A. (2015). Differences in antennal sensillae of male and female peach fruit flies in relation to hosts. *J. Insect Sci.* 15, 8-8. doi: 10.1093/jisesa/ieu178

Bay, D. E., and Pitts, C. W. (1976). Antennal olfactory sensilla of the face fly, *Musca autumnalis* Degreer (Diptera: Muscidae). *Int. J. Insect Morphol*. 5, 1-16. doi: 10.1016/0020-7322(76)90017-9

Been, T. H., Schomaker, C. H., and Thomas, G. (1988). Olfactory sensilla on the antenna and maxillary palp of the sheep head fly, *Hydrotaea irritans* (Fallen) (Diptera: Muscidae). *Int. J. Insect Morphol.* 17, 121-133. doi: 10.1016/0020-7322(88)90006-2

Bisotto-De-Oliveira, R., Redaelli, L. R., and Sant'Ana, J. (2010). Morphometry and distribution of sensilla on the antennae of *Anastrepha fraterculus* (Wiedemann) (Diptera: Tephritidae). *Neotrop. Entomol*. 40, 212-216. doi: 10.1590/s1519-566x2011000200009

de Fernandes, F. F., Pimenta, P. F., and Linardi, P. M. (2004). Antennal sensilla of the New World screwworm fly, *Cochliomyia hominivorax* (Diptera: Calliphoridae). *J. Med. Entomol.* 41, 545-51. doi: 10.1603/0022-2585-41.4.545

Gao, H., Lai, S., Zhai, Y., Lv, Z., Zheng, L., Yu, Y., et al. (2019). Comparison of the antennal sensilla and compound eye sensilla in four *Drosophila* (Diptera: Drosophilidae) species. *Fla. Entomol.* 102, 747-754. doi: 10.1653/024.102.0412

Greenberg, B., and Ash, N. (1972). Setiferous plaques on antennal pedicels of Muscoid Diptera: appearance in various species and tests of function. *Ann. Entomol. Soc. Am*. 65, 1340-1346. doi: 10.1093/aesa/65.6.1340

Hassan, M. I., Fouda, M. A., Hammad, K. M., Basiouny, A. L., and Kamel, M. R. (2013). The ultrastructure of sensilla associated with mouthparts and antennae of *Lucilia cuprina*. *J. Egyp. Soc. Parasitol*. 43, 777-785. doi: 10.12816/0006434

Henderson, D. E. H., and Wellington, W. G. (1982). Antennal sensilla of some aphidophagous Syrphidae (Diptera): fine structure and electroantennogramme study. *Can. J. Zool*. 60, 3172-3186. doi: 10.1139/z82-403

Honda, I., Ishikawa, Y., and Matsumoto, Y. (1983). Morphological studies on the antennal sensilla of the onion fly *Hylemya antiqua* MEIGEN (Diptera: Anthomyiidae). *Appl. Entomol. Zool.* 18, 170-181. doi: 10.1303/aez.18.170

Hore, G., Maity, A., Naskar, A., Ansar, W., Ghosh, S., Saha, G. K., et al. (2017). Scanning electron microscopic studies on antenna of *Hemipyrellia ligurriens* (Wiedemann, 1830) (Diptera: Calliphoridae)—A blow fly species of forensic importance. *Acta Trop.* 172, 20-28. doi: 10.1016/j.actatropica.2017.04.005

Hore, G., Saha, G. K., and Banerjee, D. (2018). Sensory organs of forensically important fly *Ophyra capensis* (Wiedemann, 1818) (Diptera: Muscidae): A scanning electron microscopic study. *Acta Trop.* 185, 400-411. doi: 10.1016/j.actatropica.2018.06.023

Hu, F., Zhang, G., Jia, F., Dou, W., and Wang, J. (2010). Morphological characterization and distribution of antennal sensilla of six fruit flies (Diptera: Tephritidae). *Ann. Entomol. Soc. Am*. 103, 661-670. doi: 10.1603/AN09170

Isaac, C., Ravaiano, S. V., Vicari Pascini, T., and Ferreira Martins, G. (2015). The antennal sensilla of species of the *Palpalis* group (Diptera: Glossinidae). *J. Med. Entomol.* 52, 614-621. doi: 10.1093/jme/tjv050

Jeong, S. A., Kim, J., Byun, B., Oh, H., and Park, K. C. (2020). Morphological and ultrastructural characterization of olfactory sensilla in *Drosophila suzukii*: scanning and transmission electron microscopy. *J. Asia-Pac. Entomol.* 23, 1165-1180. doi: 10.1016/j.aspen.2020.06.009

Li, X. Y., Liu, X. H., Ge, Y. Q., and Zhang, D. (2015). Scanning electron microscopy of antennal sensory organs of the cattle grub, *Hypoderma lineatum* (Diptera: Oestridae). *Parasitol. Res.* 114, 3865-3871. doi: 10.1007/s00436-015-4617-6

Liscia, A., Angioni, P., Sacchetti, P., Poddighe, S., Granchietti, A., Setzu, M. D., et al. (2013). Characterization of olfactory sensilla of the olive fly: Behavioral and electrophysiological responses to volatile organic compounds from the host plant and bacterial filtrate. *J. Insect Physiol.* 59, 705-716. doi: 10.1016/j.jinsphys.2013.04.008

Liu, X. H., Li, X. Y., Li, K., and Zhang, D. (2015). Ultrastructure of antennal sensory organs of horse nasal-myiasis fly, *Rhinoestrus purpureus* (Diptera: Oestridae). *Parasitol. Res.* 114, 2527-2533. doi: 10.1007/s00436-015-4453-8

Liu, X. H., Liu, J. J., Li, X. Y., and Zhang, D. (2016). Antennal sensory organs of *Scathophaga stercoraria* (Linnaeus, 1758) (Diptera: Scathophagidae): ultramorphology and phylogenetic implications. Zootaxa 4067, 361-372

Liu, Y., He, J., Zhang, R., and Chen, L. (2020). Sensilla on antenna and maxillary palp of *Neoceratitis asiatica* (Diptera: Tephritidae). *Micron* 138, 102921. doi: 10.1016/j.micron.2020.102921

Oh, H. W., Jeong, S. A., Kim, J., and Park, K. C. (2019). Morphological and functional heterogeneity in olfactory perception between antennae and maxillary palps in the pumpkin fruit fly, *Bactrocera depressa*. *Arch. Insect Biochem*. 101. doi: 10.1002/arch.21560

Pezzi, M., Whitmore, D., Chicca, M., Semeraro, B., Brighi, F., and Leis, M. (2016). Ultrastructural morphology of the antenna and maxillary palp of *Sarcophaga tibialis* (Diptera: Sarcophagidae). *J. Med. Entomol.* 53, 807-814. doi: 10.1093/jme/tjw061

Ross, K. T. A. (1992). Comparative study of the antennal sensilla of five species of root maggots: *Delia radicum* L., *D. floralis* F., *D. antiqua* Mg., *D. platura* MG. (Diptera: Anthomyiidae) and *Psila rosae* F. (Diptera: Psilidae). *Int. J. Insect Morphol.* 21, 175-197. doi: 10.1016/0020-7322(92)90015-f

Setzu, M. D., Poddighe, S., and Angioy, A. M. (2011). Sensilla on the antennal funiculus of the blow fly, *Protophormia terraenovae* (Diptera: Calliphoridae). *Micron* 42, 471-477. doi: 10.1016/j.micron.2011.01.005

Shanbhag, S. R., Müller, B., and Steinbrecht, R. A. (1999). Atlas of olfactory organs of *Drosophila melanogaster*: 1. Types, external organization, innervation and distribution of olfactory sensilla. *Int. J. Insect Morphol.* 28, 377-397. doi: 10.1016/S0020-7322(99)00039-2

Shanbhag, S. R., Singh, K., and Singh, R. N. (1995). Fine structure and primary sensory projections of sensilla located in the sacculus of the antenna of *Drosophila melanogaster*. *Cell Tissue Res*. 282, 237-249. doi: 10.1007/BF00319115

Smallegange, R. C., Kelling, F. J., and Otter, C. J. D. (2008). Types and numbers of sensilla on antennae and maxillary palps of small and large houseflies, *Musca domestica* (Diptera, Muscidae). *Microsc. Res. Techniq.* 71, 880-886. doi: 10.1002/jemt.20636

Sukontason, K., Methanitikorn, R., Chaiwong, T., Kurahashi, H., Vogtsberger, R. C., and Sukontason, K. L. (2007). Sensilla of the antenna and palp of *Hydrotaea chalcogaster* (Diptera: Muscidae). *Micron* 38, 218-223. doi: 10.1016/j.micron.2006.07.018

Sukontason, K., Sukontason, K. L., Piangjai, S., Boonchu, N., Chaiwong, T., Ngern-Klun, R., et al. (2004). Antennal sensilla of some forensically important flies in families Calliphoridae, Sarcophagidae and Muscidae. Micron 35, 671-9. doi: 10.1016/j.micron.2004.05.005

Tangtrakulwanich, K., Chen, H., Baxendale, F., Brewer, G., and Zhu, J. J. (2011). Characterization of olfactory sensilla of *Stomoxys calcitrans* and electrophysiological responses to odorant compounds associated with hosts and oviposition media. *Med. Vet. Entomol.* 25, 327-36. doi: 10.1111/j.1365-2915.2011.00946.x

Wang, Q., Liu, X., Lu, P., and Zhang, D. (2014b). Ultrastructure of antennal sensilla in *Hydrotaea armipes* (Diptera: Muscidae): New evidence for taxonomy of the genus *Hydrotaea*. *Zootaxa* 3790, 577-586. doi: 10.11646/zootaxa.3790.4.6

Wang, Q., Yang, Y., Liu, M., and Zhang, D. (2014a). Fine structure of *Delia platura* (Meigen) (Diptera: Anthomyiidae) revealed by scanning electron microscopy. *Microsc. Res. Techniq*. 77, 619-630. doi: 10.1002/jemt.22380

Wang, Q., Zhang, M., Li, K., and Zhang, D. (2012). Olfactory sensilla on antennae and maxillary palps of *Fannia hirticeps* (Stein, 1892) (Diptera: Fanniidae). *Microsc. Res. Techniq.* 75, 1313-1320. doi: 10.1002/jemt.22066

Zhang, D., Li, X., Liu, X., Wang, Q., and Pape, T. (2016). The antenna of horse stomach bot flies: morphology and phylogenetic implications (Oestridae, Gasterophilinae: *Gasterophilus* Leach). *Sci. Rep.* 6, 34409. doi: 10.1038/srep34409

Zhang, D., Liu, X. H., Li, X. Y., Cao, J., Chu, H. J., and Li, K. (2015). Ultrastructural investigation of antennae in three cutaneous myiasis flies: *Melophagus ovinus*, *Hippobosca equina*, and *Hippobosca longipennis* (Diptera: Hippoboscidae). *Parasitol. Res.* 114, 1887-1896. doi: 10.1007/s00436-015-4376-4

Zhang, D., Liu, X. H., Li, X. Y., Zhang, M., and Li, K. (2013c). Antennal sensilla of the green bottle fly, *Lucilia sericata* (Meigen) (Diptera: Calliphoridae). *Parasitol. Res.* 112, 3843-3850. doi: 10.1007/s00436-013-3573-2

Zhang, D., Liu, X. H., Wang, Q. K., and Li, K. (2014). Sensilla on the antenna of blow fly, *Triceratopyga calliphoroides* Rohdendorf (Diptera: Calliphoridae). *Parasitol. Res.* 113, 2577-2586. doi: 10.1007/s00436-014-3909-6

Zhang, D., Wang, Q. K., Hu, D. F., and Li, K. (2012). Sensilla on the antennal funiculus of the horse stomach bot fly, *Gasterophilus nigricornis*. *Med. Vet. Entomol*. 26, 314-322. doi: 10.1111/j.1365-2915.2011.01007.x

Zhang, D., Wang, Q. K., Yang, Y. Z., Chen, Y. O., and Li, K. (2013a). Sensory organs of the antenna of two Fannia species (Diptera: Fanniidae). *Parasitol. Res.* 112, 2177-2185. doi: 10.1007/s00436-013-3377-4

Zhang, D., Wang, Q., Liu, X., and Li, K. (2013b). Sensilla on antenna and maxillary palp of predaceous fly, *Lispe neimongola* Tian et Ma (Diptera: Muscidae). *Micron* 49, 33-39. doi: 10.1016/j.micron.2013.02.012
